# Face processing in 22q11.2 deletion syndrome: atypical development and visual scanning alterations

**DOI:** 10.1186/s11689-018-9245-x

**Published:** 2018-08-29

**Authors:** Alexandra Zaharia, Maude Schneider, Bronwyn Glaser, Martina Franchini, Sarah Menghetti, Marie Schaer, Martin Debbané, Stephan Eliez

**Affiliations:** 1 Developmental Imaging and Psychopathology Lab, Department of Psychiatry, University of Geneva School of Medicine, Geneva, Switzerland; 2 Swiss Center for Affective Sciences, University of Geneva, Geneva, Switzerland; 3 Center for Contextual Psychiatry, Research Group Psychiatry, Department of Neurosciences, KU Leuven, Leuven, Belgium; 4 Stanford Cognitive and Systems Neuroscience Laboratory, Stanford University School of Medicine, California, USA; 5 Adolescence Clinical Psychology Research Unit, Faculty of Psychology and Educational Sciences, University of Geneva, Geneva, Switzerland; 6 Department of Genetic Medicine and Development, University of Geneva, Geneva, Switzerland

**Keywords:** Configural face processing, Featural face processing, Eye-tracking, Neurodevelopmental disorders, Social difficulties

## Abstract

**Background:**

Previous research links social difficulties to atypical face exploration in 22q11.2 deletion syndrome (22q11.2DS). Two types of face processing are distinguished: configural (CFP) and featural (FFP). CFP develops later in life and plays an important role in face and emotion recognition abilities. Recent studies reported atypical development of CFP in several neurodevelopmental disorders. Taking previous reports of atypical face exploration one step further, our study aims at characterizing face processing in children and adolescents with 22q11.2DS. First, we sought to identify biases in the first two fixation positions on faces and to detect differences between CFP and FFP in 22q11.2DS using eye-tracking technology. Second, we investigated the developmental trajectories of CFP and FFP using accuracy data from follow-up evaluation.

**Methods:**

Seventy-five individuals with 22q11.2DS and 84 typically developed (TD) individuals (aged 6–21 years) completed a discrimination task (“Jane task”) inducing CFP and FFP in an eye-tracking setting. Thirty-six individuals with 22q11DS and 30 TD from our sample completed a longitudinal follow-up evaluation.

**Results:**

Findings revealed that individuals with 22q11.2DS demonstrate an early bias toward the mouth region during the initial fixations on the faces and reduced flexibility exploration of the faces, with a reduced number of transitions between faces and longer fixations compared to the TD group. Further, scanpaths did not differ between CFP and FFP in the 22q11.2DS group. Longitudinal analysis of accuracy data provided evidence for atypical development of CFP in 22q11.2DS.

**Conclusions:**

The current study brings new evidence of altered face exploration in 22q11.2DS and identifies developmental mechanisms that may contribute to difficulties impacting social interactions in the syndrome.

## Background

22q11.2 deletion syndrome (22q11.DS), also known as DiGeorge or velocardiofacial syndrome, is one of the most frequent microdeletions, with an incidence of approximately 1:2000 in pregnancies and 1:4000 births [1–3]. The syndrome has been associated with a characteristic facial appearance, hypernasal speech, cardiac anomalies, learning disabilities, attention deficits, and social impairments [4, 5]. Specifically, the 22q11.2DS social phenotype is characterized by social and emotional withdrawal, high rates of shyness and anxiety disorders, and difficulties initiating and maintaining social interactions [4–9]. Studies on other clinical populations with social dysfunction such as Down and Williams syndromes, autism spectrum disorder (ASD), developmental prosopagnosia, and schizophrenia have highlighted the implications of face processing skills for social interaction [10–12]. The investigation of face processing was also found to be highly relevant to gain a better insight into the mechanisms associated with social difficulties in 22q11.2DS [13, 14]. Several studies using eye-tracking technology have reported alterations of visual scanpaths during face and emotion processing in small samples of children and adolescents with 22q11.2DS compared to control groups [13–16]. The mean age of individuals participating in these studies ranged from 12.36 to 17.4 years old. When looking at non-emotional/neutral or emotional faces, children and adolescents with 22q11.2DS fixated more on the mouth and less on the eyes than typically developed (TD) and developmentally delayed groups and tended to have fewer fixations and shorter scanpath lengths than controls [13, 14]. Other studies on emotional face processing showed that adolescents with 22q11.2DS fixated less on face stimuli than controls, which is reminiscent of results in individuals with ASD [15, 17]. Finally, McCabe et al. [17] argue that a failure to change exploration patterns according to the content of the visual information (i.e., perseverative and inflexible behavior) may play a role in the aberrant pattern of fixations on faces in 22q11.2DS. These eye-tracking findings were examined in relation to several aspects of the 22q11.2DS phenotype in order to understand their relationship with social difficulties. In particular, Glaser et al. [13] observed a significant negative association between time spent on the eyes and higher rates of anxiety, which could suggest a link between impairments related to the processing of socially relevant stimuli and the socio-emotional dysfunctions found in 22q11.2DS.

The ability to process faces improves with age and is associated to the comprehension of emotional and mental states and to an adequate communication and behavior during social interactions [12, 18, 19]. Previous studies have differentiated two core mechanisms of visual scanning of faces: configural face processing (CFP) and featural face processing (FFP) [20, 21]. FFP (or component processing) refers to the exploration of individual parts of a face, such as contour, color, and shape of the facial features (e.g., nose, eyes, mouth). On the other hand, CFP refers to the analysis of spatial distances between the features. CFP contributes to the achievement of a high level of expertise in face recognition and in emotion recognition [22–24]. From a developmental perspective, CFP develops significantly later than FFP [25, 26]. Whereas some studies indicate that CFP is already adult-like by the age of 10 [25, 26], others suggest that we reach proficiency in CFP in adulthood only [11, 19]. Although CFP and FFP show different developmental trajectories, together they account for the expert skills observed in adulthood [19, 27].

Evidence for atypical development of CFP has been reported in several populations with social impairments. In order to measure CFP and FFP, face discrimination tasks were administrated to participants [26]. In these tasks, faces were modified according to the specific type of face processing: configural changes affected the distances between features and featural changes included differences in features without manipulating the distances between them (e.g., replacing the eyes with the ones of another person). Lower accuracy in discriminating faces with configural changes has been reported in children with neurodevelopmental disorders (e.g., ASD, Williams and Down syndromes) compared to TD participants [11]. This observation suggests that the development of CFP is fragile and easily altered in neurodevelopmental disorders. Nonetheless, high-functioning adults with ASD were shown to have similar CFP compared to controls, which reinforces the usefulness of examining CFP from a developmental perspective [28]. To our knowledge, only one study explored FFP and CFP in 22q11.2DS and demonstrated impairments and lack of improvement with age in both types of processing [13]. To gain further insight into the development of CFP and FFP, it is necessary to investigate these difficulties in a broader age range and verify whether they expand through adulthood by using longitudinal designs.

Eye-tracking technology is a promising tool used to gather valuable information, such as eye gaze, which is not easily observable by experimenters. Hence, eye-tracking collects data regarding “when” (temporal) and “where” (spatial) the attention was allocated to a stimulus, such as the number of fixations, percentage of time spent, fixation duration, location of first fixations, and number of transitions between areas of interest [29]. Previous studies [30–32] also recommended examining biases that could occur during the early phase of information processing (e.g., the landing positions of first and second fixations, the sequence of first fixations). Accordingly, the initial fixation positions could influence the pattern of fixations that will follow during visual scanning and are important for an optimal information extraction. For instance, it was proven that the first two fixations suffice to achieve performance in a face-recognition task [32]. Furthermore, recent eye-tracking studies revealed that individuals with disorders were less likely to return to the eyes region during face scanning and more likely to hyperscan the remaining facial features [33, 34].

To date, no study has used combined measures of accuracy in discriminating faces with configural and featural changes and eye-tracking to explore CFP and FFP, along with their development over time in 22q11.2DS. In the present study, a large sample of participants with 22q11.2DS and TD with a wide age range performed a face discrimination task (“Jane task”), in which participants are presented with two portraits with variations in the individual features (FFP) or spacing of the features (CFP) and asked to judge whether they are identical or different [13, 26]. Our first aim was to extend the results previously described by Glaser et al. [13] on an overlapping sample and to further characterize face processing alterations in 22q11.2DS. Taking into consideration the meaningfulness of first and second fixations for information processing [30–32], we specifically examined the first two landing positions (taken separately) in both groups. We expected to observe the presence of markers indicating an atypical face processing that could occur at the beginning of visual exploration. We therefore hypothesized that participants with 22q11.2DS would show a reduced tendency to look at the eyes during both first and second fixations on faces compared to TD participants. Based on previous evidence on perseverative visual exploration [13, 17], we also expected to observe fewer transitions between the faces and longer fixations in participants with 22q11.2DS. Our second aim was to compare visual scanning patterns during FFP and CFP. Given that difficulties found in clinical populations depend on the type of face processing (CFP or FFP) [11, 17], we hypothesized that participants from both groups would show different patterns during the exploration of faces modified on a configural or featural level. Therefore, we expected that eye movements would adapt to the type of face processing induced by the stimuli only in TD group during face exploration. Thirdly, we investigated changes in CFP and FFP occurring from childhood through adolescence using longitudinal data available in a subsample of participants. Based on the atypical development of face processing found in individuals with neurodevelopmental disorders [11, 13], we expected to observe a lack of improvement with age in the ability to discriminate configural differences in participants with 22q11.2DS. Finally, we examined whether alterations in discriminating configural and featural differences would relate to the presence of social difficulties in participants with 22q11.2DS. Specifically, we expected to observe significant associations between CFP abilities and clinical measures of social impairment (anxiety, emotional and social withdrawal, poor socialization).

## Methods

### Participants

Seventy-five individuals with 22q11.2DS and 84 TD individuals (comparison group) were included. Participants ranged from 6 to 21 years old. Descriptive characteristics for both groups are reported in Table 1. The two groups did not differ by age (*t*(157) = .11, *p* = .91, *d* = .019) or gender (χ^2^_(1, 159)_ = .18, *p* = .68). One of the inherent characteristics of individuals with 22q11.2DS is lower IQ compared to the general population [35], which can also be observed in our sample (Table 1). Therefore, the two groups were not matched on IQ. Participants with 22q11.2DS were recruited through parent associations and word of mouth, while the comparison group included siblings as well as individuals recruited in the public schools and through announcements in the Geneva community. During recruitment, all participants were screened for lifetime history of psychiatric and neurological conditions during an interview with a trained psychiatrist (SE) in order to determine their initial eligibility in the study. Participants were tested in our research unit for an ongoing longitudinal study. All participants signed written consent forms approved by local ethical review board. Data from 25 (33.33%) individuals with 22q11.2DS and 21 (26.19%) TD participants were also included in a previously published study using the same experimental paradigm [13].

**Table 1:** Demographic characteristics

	Typically developed		22q11.2DS	
	Cross-sectional/longitudinal (T1)	Longitudinal (T2)	Cross-sectional/longitudinal (T1)	Longitudinal (T2)
*N*	84	30	75	36
Gender ratio (females/males)	42/42	17/13	40/35	19/17
Age (years)	12.88 (3.9)	16.4 (3.43)	12.81(3.57)	16.45 (3.09)
Intellectual functioning				
Full Scale IQ	111.32 (13.68)	106.60 (11.63)	71.96 (11.25)	69.06 (11.62)
Verbal IQ	111.67 (12.96)	106.37 (11.39)	78.30 (13.69)	72.75 (13.31)
Performance IQ	108.06 (15.34)	105.37 (13.75)	71.22 (11.27)	70.14 (11.38)
Perceptual Organization Index	108.60 (15.39)	104.27 (13.24)	71.97 (11.48)	71.25 (11.41)
Processing Speed Index	107.78 (15.84)	108.70 (12.34)	82.54 (16.23)	77.86 (16.20)
ABCL/CBCL (T scores)	Available for 82/84	Available for 29/30	Available for 75/75	Available for 36/36
Internalizing	47.72 (9.63)	46.52 (8.68)	63.52 (11.76)	62.50 (10.83)
Anxiety-Depression	52.98 (5.43)	52.45 (5.75)	62.13 (10.57)	61.14 (10.03)
PANSS (T scores)			Available for 63/75	Available for 36/36
Negative	–	–	48.24 (12.23)	49.78 (14.02)
Positive	–	–	37.46 (8.19)	38.14 (7.32)

The presence of a 22q11.2 microdeletion was confirmed using quantitative fluorescent polymerase chain reaction. Participants with 22q11.2DS were screened for psychiatric disorders using the Diagnostic Interview for Children and Adolescents—Revised (DICA-R) [36] and the psychosis supplement of Schedule for Affective Disorders and Schizophrenia for School-Age Children-Present and Lifetime Version (Kiddie-SADS-P/L) [37] for the participants younger than 18 years old and the Structured Clinical Interview for DSM-IV axis I disorders (SCID-I) [38] for participants older than 17 years old. A trained psychiatrist (SE) conducted the structured diagnostic interviews. The psychiatric disorders present in our sample, in descending order of frequency, were as follows: specific phobias (46.67%), attention-deficit/hyperactivity disorder (ADHD, 25.33%), generalized anxiety disorder (17.33%), major depressive disorder (9.33%), social phobia (8%), psychotic disorder (8%), oppositional defiant disorder (5.33%), and obsessive-compulsive disorder (4%). Seventeen (22.67%) individuals with 22q11.2DS were receiving psychotropic medication at testing: 10 (13.33%) were using methylphenidate, 2 (2.67%) antidepressants, 5 (6.67%) antipsychotics, and 4 (5.33%) anticonvulsants.

### Materials

#### Face discrimination task

**Fig. 1:**
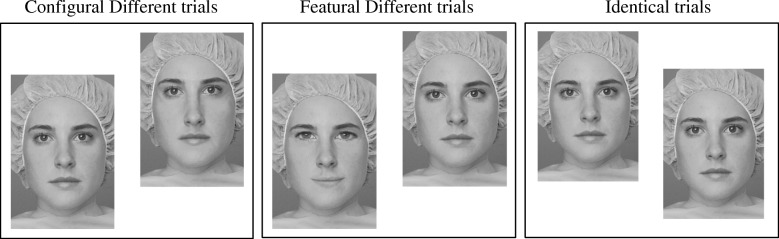
Example of possible trials during the face discrimination task (Jane task)

The Jane task [26] is a free-viewing discrimination task during which participants have to decide whether simultaneously presented photos of faces are identical or different. The stimuli consisted of eight portraits derived from a black and white original photo of a woman called “Jane.” The other eight portraits (“Jane’s sisters”) were photos that have been manipulated either configurally or featurally. Our eye-tracking Jane task consisted of 30 trials displaying photos from the configural set and 30 trials with photos from the featural set. For each set, half of the trials consisted of pairs of identical photos (either Jane or her sisters). The photos were displayed off-set in order to oblige the viewer to explore the entire structure of the face rather than detect differences based on the alignment of the stimuli. Examples from each condition: Configural Different (CD), Featural Different (FD), and Identical are presented in Fig. 1. The CD trials included the original Jane photo and four new versions of Jane that have been modified in terms of the spacing between the facial features (the new portraits included one of the four following changes: the eyes or the mouth are moved either up or down; the eyes are either closer together or further apart). The FD trials included the original Jane photo and four new portraits where the eyes and mouths have been replaced with those from other persons. This resulted in four new portraits with both eyes and mouth regions changed compared to the original. The new features had the same length as in the original portrait in order to minimize the impact that this could have on spacing among features. The validation of the stimuli is described in Mondloch et al. [26] and several studies demonstrated how CFP and FFP can be reliably examined using this paradigm [25, 39].

Each trial was preceded by a fixation cross (200 ms). The two portraits remained on the screen until the participant decided whether the portraits were identical (“press green button”) or different (“press red button”). Participants received the following standardized instructions: “You will see two portraits. Please look at them carefully and decide if the portraits are identical or different. The differences might be very subtle”. Then, they completed several practice trials. The exposure time to each stimulus, which corresponds to the reaction time, was therefore different across trials. Table 2 summarizes the average exposure time to stimuli per condition for each group at time 1. Within each set, the trials were presented in a fixed randomized order and the positions of the two portraits on the screen (up or down) were also randomly swapped. The stimuli were presented on a white background.

**Table 2:** Exposure time (ms) to stimuli: group averages and standard deviations

	TD (*N* = 84)	22q11.2DS (*N* = 75)
Condition		
FD	2093.56 (827.05)	3118.91 (1266.78)
CD	4088.47 (2349.84)	4880.57 (2522.20)
Identical	3807.39 (1804.45)	4281.72 (2255.43)

**Fig. 2:**
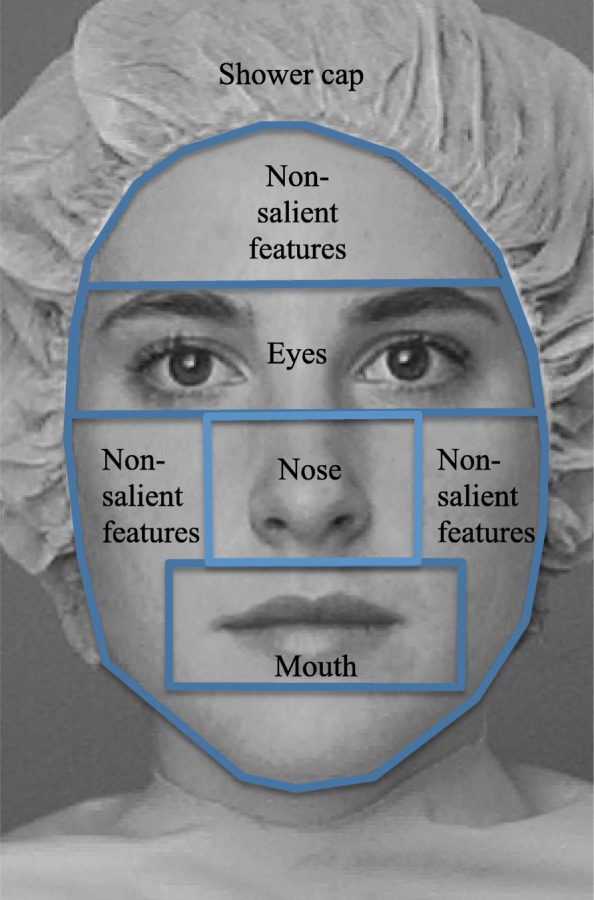
The original “Jane” photo with labeled areas of interest

The task was created in Clearview 2.7.1 software (www.tobii.com) and administered on a Tobii 1750 eye tracker with a 17-inch display, 1280 × 1024 resolution, and a sampling rate of 50 Hz. The participants were positioned at a distance of approximately 60 ± 10 cm from the screen. A five-point calibration procedure was completed before the task to ensure that participants’ eye positions on the screen could be captured. According to eye-tracking studies [40, 41] and the Tobii 1750 settings, a gaze point lasting at least 100 ms and falling within a circle encompassing 30 pixels was counted as a fixation point. To examine the visual exploration patterns for specific areas of the face, we divided the stimuli into five areas of interest (AOI, see Fig. 2). The eyes, mouth, and nose regions corresponded to the AOI size of those used in a previous study [13]. The non-salient features referred to cheeks, forehead, and chin taken together. We examined the following visual scanning parameters used in previous studies: percentages of time spent and fixation count (calculated by dividing the time or number of fixations on an AOI by the total time spent or the total number of fixations, respectively, on the entire slide containing the two portraits) and average fixation duration. Additionally, we included the locations of the first and second fixations and the number of transitions between the faces.

#### Cognitive assessment

Participants younger than 17 years old completed the Wechsler Intelligence Scale for Children—Version III (WISC-III) [42], whereas older participants completed the Wechsler Adult Intelligence Scale—Version III (WAIS-III) [43]. Full Scale Intelligence Score (FSIQ), Perceptual Organization Index (POI), Processing Speed Index (PSI), Verbal (VIQ), and Performance (PIQ) scores were available for all participants.

#### Clinical assessment

Depending on a participant’s age, parents of all participants completed the Child Behavior Checklist (CBCL) [44] or the Adult Behavior Checklist (ABCL) [45]. Parents of two TD individuals were not available to complete the questionnaires. We used the Internalizing and Anxious-Depressed T scores that were the only measures available for all age ranges to assess social difficulties such as withdrawn and/or anxiety-depression symptoms. None of the TD participants obtained scores above the clinical range on the anxiety scale.

To examine markers of social difficulties, only 63 individuals with 22q11.2DS (84% of the total sample, age range 6–22 years old) were available to take part in clinical interviews during which a trained psychiatrist administered the Positive and Negative Syndrome Scale (PANSS) [46]. This scale is composed of positive, negative, and general psychopathology subscales. Given that poor social abilities are mostly reflected in measures of negative symptoms [47], it was used as the subscale of interest. The PANSS negative symptom subscale includes the following items: blunted affect, emotional withdrawal, poor rapport, passive/apathetic social withdrawal, difficulty in abstract thinking, lack of spontaneity and flow of conversation, and stereotyped thinking.

### Data analysis

#### Cross-sectional analysis

We conducted repeated measures MANOVAs to examine interaction effects. For post-hoc comparisons, we used paired-samples *t* tests to examine within-group differences and independent-samples *t* tests to explore between-group differences. To decrease the probability of false positives, we applied a Bonferroni correction to our *p*-values by dividing the standard *p*-value by the number of comparisons performed on each test.

#### Longitudinal analysis

From the cross-sectional sample (*N*_22q11.2_ = 75, *N*_TD_ = 84), 43% of the clinical sample (*N*_22q11.2_ = 36) and 48% of the TD sample (*N*_TD_ = 30) participated in a second visit (see Table 1). The mean interval between T1 and T2 visits in these participants was 3.68 (range: 2.56 to 5.54 years). In order to maximize the number of observations, the analyses examining the developmental trajectories of CFP and FFP were performed on all available time points (i.e., data collected in participants who completed only T1 assessment and participants who completed T1 and T2 assessments) using mixed regression analyses.

Briefly, the within-subjects factor was introduced as a nested variable to increase our statistical power in a longitudinal dataset using a variable time interval between the visits and a broad age range. Using the nlmefit function in MATLAB R2011b (MathWorks), several intercept models were proposed (constant, linear, quadratic, and cubic). A Bayesian information criterion (BIC)-based model selection method was selected for the power it gives mixed models. Next, a likelihood ratio test was used to quantify significant between-group differences in face processing trajectories over time. Given that, two types of differences can be observed between groups: shape differences for the developmental trajectories and intercept differences (i.e., the two trajectories have the same shape but not the same intercept) between the two groups. The statistical approach has been described in detail elsewhere [48, 49].

## Results

### Eye-tracking measures (cross-sectional sample)

#### Descriptive data

One TD and five participants with 22q11.2DS did not present satisfactory eye-tracking quality due to frequent gaze fluctuations and had to be excluded from the analyses (each gaze recording was manually verified and participants for whom the eye tracker failed at collecting any data were excluded). Hence, the results reported in this section were based on a sample of 70 participants with 22q11.2DS and 83 TD individuals. The results described below remained unchanged when individuals with 22q11.2DS who were receiving psychotropic medication (*N* = 17) were excluded from the analyses.

#### Time spent and fixations on AOI

**Fig. 3:**
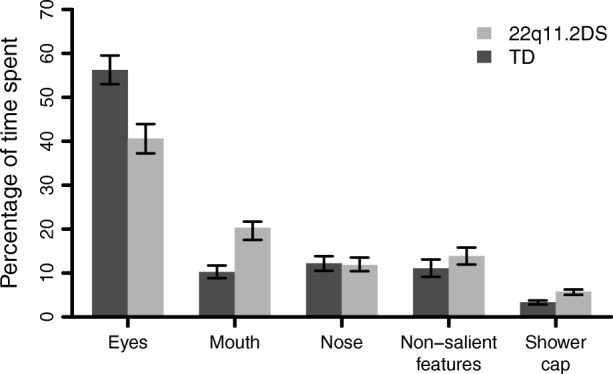
Percentage of time spent on each AOI in TD and 22q11.2DS groups. Standard errors are represented in the figure by the error bars attached to each column

A 2 × 5 repeated measures MANOVA revealed a significant interaction effect between group and time spent on AOI (eyes, mouth, nose, non-salient features, and shower cap): *F*(4, 148) = 10.77, *p* < .001, *η*_*p*_^2^ = .23 (see Fig. 3). Post-hoc tests showed that 22q11.2DS group spent significantly less time on eyes than the comparison group (*M*_22q11.2DS_ = 0.40, *SD*_22q11.2DS_ = 0.22, *M*_TD_ = 0.56; *SD*_TD_ = 0.21, *t*(144.48) = − 4.49, *p* < .001). However, they spent significantly more time on mouth (*M*_22q11.2DS_ = 0.20, *SD*_22q11.DS_ = 0.17; *M*_TD_ = 0.10, *SD*_TD_ = 0.09, *t*(100.31) = 4.29, *p* < .001) and on shower cap regions (*M*_22q11.2DS_ = 0.06, *SD*_22q11.DS_ = 0.05; *M*_TD_ = 0.03, *SD*_TD_ = 0.03, *t*(109.93) = 3.83, *p* < .001) than the comparison group. Within-group analyses showed that the percentage of time spent on eyes was significantly higher than the percentage of time spent on mouth in both groups (22q11.2DS: *t*(69) = 4.72; *p* < .001; TD: *t*(82) = 15.23, *p* < .001).

A significant interaction effect was also found when we ran the same analysis for the number of fixations (*F*(4, 148) = 12.05, *p* < .001, *η*_*p*_^2^ = .25). The post-hoc tests revealed the same significant differences between and within group as we showed above for the percentage of time spent on AOI (*p* < .001).

#### First and second fixations

**Fig. 4:**
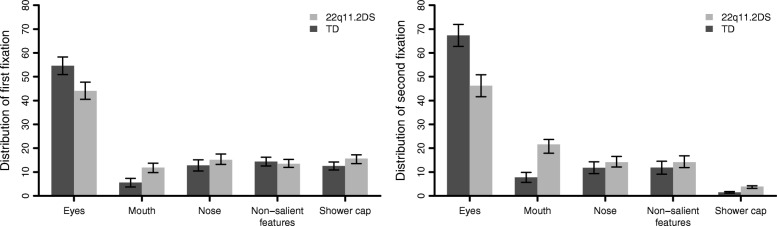
Percentage of first and second fixations on each AOI in TD and 22q11.2DS groups. Standard errors are represented in the figure by the error bars attached to each column

A 2 × 2 × 5 repeated measures MANOVA showed a significant interaction between group, the fixation (first, second) and the AOI (eyes, mouth, nose, non-salient features, shower cap): *F*(4, 151) = 6.22, *p* < .001, *η*_*p*_^2^ = .14. Post hoc tests revealed that the 22q11.2DS group showed a significantly lower percentage of first and second fixations on the eye region (first fixation: *M*_22q11.2DS_ = 0.44, *SD*_22q11.DS_ = 0.23; *M*_TD_ = 0.55, *SD*_TD_ = 0.24, *t*(148.62) = 2.84, *p* = .005; second fixation: *M*_22q11.2DS_ = 0.46, *SD*_22q11.DS_ = 0.29; *M*_TD_ = 0.67, *SD*_TD_ = 0.30, *t*(147.29) = 4.43, *p* < .001) than TD group. They also showed a significantly greater percentage of first and second fixations on the mouth region (first fixation: *M*_22q11.2DS_ = 0.12, *SD*_22q11.DS_ = 0.14; *M*_TD_ = 0.05, *SD*_TD_ = 0.12, *t*(136.99) = − 3.04, *p* = .003; second fixation: *M*_22q11.2DS_ = 0.22, *SD*_22q11.DS_ = 0.24; *M*_TD_ = 0.07, *SD*_TD_ = 0.14, *t*(105.99) = − 4.31, *p* < .001) and of the second fixations on the shower cap region (*M*_22q11.2DS_ = 0.04, *SD*_22q11.DS_ = 0.05; *M*_TD_ = 0.02, *SD*_TD_ = 0.02, *t*(93.04) = − 3.86, *p* < .001) compared to TD individuals. Within-group comparisons revealed that the percentage of second fixations on the eyes was significantly higher than the percentage of first fixations on the eyes in the TD group (*t*(82) = − 7.67, *p* < .001). By contrast, the percentage of second fixations on the mouth region was higher than the first fixations on the mouth in the 22q11.2DS group (*t*(69) = − 5.99, *p* < .001). As for the eye region in 22q11.2DS and mouth in TD, there were no significant differences between the percentage of first and second fixations. The distribution of first and second fixations on the five AOI is presented in detail in Fig. 4.

#### Transitions and average fixation duration

The comparison group made significantly more transitions between the two portraits presented on the screen than individuals with 22q11.2DS (*M*_22q11.2DS_ = 177.51, *SD*_22q11.DS_ = 87.81; *M*_TD_ = 221.22, *SD*_TD_ = 93.76, *t*(149.32) = − 2.97, *p* = .003). Average fixation duration (ms) was longer in individuals with 22q11.2DS than in the comparison group (*M*_22q11.2DS_ = 295.21, *SD*_22q11.DS_ = 67.83; *M*_TD_ = 248.79, *SD*_TD_ = 44.39, *t*(115.21) = 4.91, *p* < .001).

#### Visual scanpath during CFP versus FFP

Two separated 2 × 2 × 3 repeated measures MANOVAs on the percentage of time spent and number of fixations indicated a significant interaction between conditions (CD, FD), the time spent on AOIs (eyes, mouth, nose), and group (*F*(2, 150) = 5.60, *p* = .005, *η*_*p*_^2^ = .07, respectively *F*(2, 150) = 5.65, *p* = .004, η_p_^2^ = .07). Post-hoc analyses indicated that 22q11.2DS spent less time and made fewer fixations on eyes and more time and more fixations on mouth than TD group across conditions. However, paired-samples *t* tests indicated that only the TD group showed significant differences in visual exploration between CD and FD trials: they spent more time and made more fixations on eyes in FD than in CD and spent more time and made more fixations on mouth in CD than FD. Scanpath characteristics and detailed analyses for CD and FD items in both groups are summarized in Table 3.

**Table 3:** Within-group differences between Featural Different (FD) and Configural Different (CD) trials during face scanning

	TD (*N* = 83)			22q11.2DS (*N* = 70)		
	*M*(*SD*)		*t*-value	*M*(*SD)*		*t*-value
	FD	CD		FD	CD	
Time spent (%)						
Eyes	62.93 (24.72)	55.69 (23.01)	4.15*	41.94 (27.40)	39.56 (21.39)	1.18
Mouth	6.28 (9.44)	10.58 (11.74)	− 5.41*	23.54 (23.66)	18.89 (17.74)	2.25
Nose	8.40 (11.32)	12.59 (12.66)	− 4.71	11.27 (11.89)	11.81 (10.18)	− 0.42
Number of fixations (%)^1^						
Eyes	59.80 (22.95)	53.20 (21.33)	4.07*	39.94 (24.21)	37.50 (18.57)	1.39
Mouth	6.02 (8.89)	10.42 (10.81)	− 6.02*	21.27 (20.28)	17.70 (15.04)	2.03
Nose	8.72 (11.16)	12.63 (11.34)	− 4.56	11.97 (10.49)	12.23 (9.01)	− 0.24

*significant at *p* < .001; ^1^Percentage out of the total number of fixations on the entire slide during the target condition

### Accuracy on the task

#### Cross-sectional analyses

**Fig. 5:**
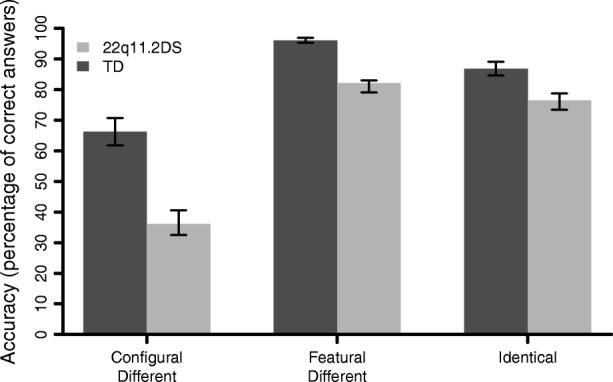
Mean accuracy scores (percentage of correct answers) for the three conditions of Jane task. Standard errors are represented in the figure by the error bars attached to each column

A 2 × 3 repeated measures MANOVA was conducted to compare scores on the percentage of correct answers (accuracy) across conditions (CD, FD, Identical) between groups. We found a significant interaction effect between group and condition (*F*(2,156) = 7.66, *p* = .001, *η*_*p*_^2^ = .09). Post-hoc comparisons indicated that the comparison group performed better than the 22q11.2DS group across all three conditions (*p* < .001; see Fig. 5): CD (*M*_TD_ = 0.66, *SD*_TD_ = 0.29; *M*_22q11.2DS_ = 0.36; *SD*_22q11.2DS_ = 0.22, *t*(154.79) = 7.39), FD (*M*_TD_ = 0.96, *SD*_TD_ = 0.05; *M*_22q11.2DS_ = 0.82, *SD*_22q11.2DS_ = 0.19, *t*(84.25) = 5.93), and Identical trials (*M*_TD_ = 0.86, *SD*_TD_ = 0.15; *M*_22q11.DS_ = 0.76; *SD*_22q11.2DS_ = 0.19, *t*(134.74) = 3.74). Moreover, the gap between CD and FD accuracy performance was significantly larger in individuals with 22q11.2DS compared to TD (*M*_TD_ = − 0.29, *SD*_TD_ = 0.27; *M*_22q11.DS_ = − 0.46; *SD*_22q11.2DS_ = 0.26, *t*(155.72) = 3.83, *p* < .001). Paired-samples *t* tests showed significant within-group differences (*p* < .001) between all the three conditions for the comparison group (FD > CD, *t*(83) = 10.15; Identical > CD, *t*(83) = 5.65; FD > Identical, *t*(83) = 5.64). In the 22q11.2DS group, accuracy performances were significantly different (*p* < .001) only between FD and CD (FD > CD, *t*(74) = 15.16) and Identical and CD trials (Identical > CD, *t*(74) = 9.74), whereas the difference between FD and Identical trials accuracy was not significant (*t*(74) = 1.93, *p* = .058). These results did not differ when individuals with 22q11.2DS diagnosed with a psychotic disorder (*N* = 6) were excluded from the analyses.

Paired-samples *t* tests also showed that both groups made more errors during the Different trials taken together (answered “identical” when faces were different) than during the Identical trials (answered “different” when faces are identical): *t*(74) = − 5.34, *p* < .001 (22q11.2DS group); *t*(83) = − 2.35, *p* = .021 (TD group).

#### Longitudinal analysis on developmental trajectory of face processing abilities

**Fig. 6:**
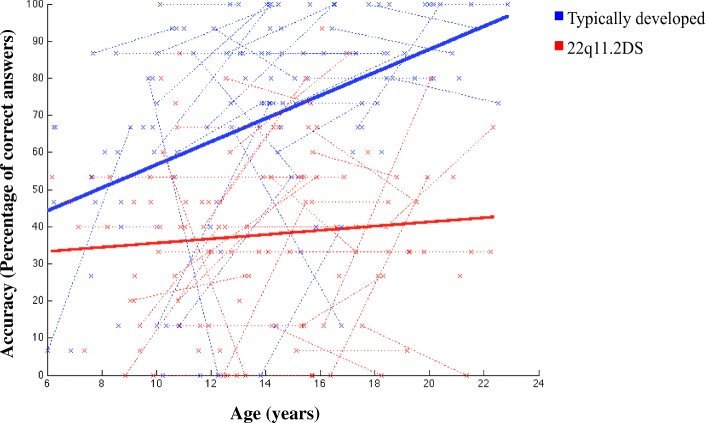
Developmental trajectory of configural face processing in TD and 22q11.2DS groups

Mixed model regression analysis showed a significant difference in the shape of the trajectories between the two groups (*N*_22q11.2DS and TD_ = 225, *p* = .0028; see Fig. 6) in CD trials.

In both groups, the trajectories were linear but increased less drastically with age in participants with 22q11.2DS compared to TD individuals. Hence, accuracy on CD trials significantly increased with age in the TD group but not in the 22q11.2DS group. Due to ceiling effects observed in both groups, the developmental curves of FD and Identical trials were not interpretable.

### Associations with clinical measures of social difficulties

No significant correlation was observed between IQ scores and eye-tracking or behavioral measures when running within-group correlation analyses. Additionally, no significant associations were found between ABCL-CBCL measures (internalizing and anxious-depressed scales) and any eye-tracking or task performance measures (for instance, there was no significant correlation between visual scanpath measures and accuracy on the task). However, Pearson correlations revealed a significant negative correlation between the percentage of correct answers on CD trials and the PANSS negative score (*r* = −.293, *p* = .02) in the 22q11.2DS sample.

## Discussion

The current study replicated previous findings [13] and covered new aspects regarding the face processing in 22q11.2DS in a longitudinal sample. Hence, we showed that difficulties in face processing persist throughout a broad age range of individuals with 22q11.2DS, from childhood to adulthood. Particularly, participants with 22q11.2DS showed a bias toward the mouth region during the first two fixations on faces and an increased number of fixations toward this specific region from the first to the second fixation. They also had longer fixations and spent more time looking at less relevant regions. This pattern is indicative of a perseverative type of scanning which could impair flexibility and the identification of relevant information during visual exploration of human faces. Participants with 22q11.2DS did not show distinct visual scanpaths between CFP and FFP, whereas the TD group used different eye scanning strategies depending on the type of processing. We also showed that CFP accuracy improved less drastically with age in the 22q11.2DS group, compared to the TD group.

The first aim of our study was to delineate face scanning in 22q11.2DS using various eye-tracking parameters: first and second fixation locations, average fixation duration, number of transitions during face exploration, and scanpath patterns during CFP versus FFP. First, we found a higher proportion of first fixations on the mouth in the 22q11.2DS group compared to the TD group and a higher proportion on the eyes in the TD group compared to the 22q11DS group. Unlike the comparison group, we did not observe an increase in the number of fixations on the eye region from the first to the second fixation in participants with 22q11DS. Rather, the 22q11.2DS group increased their fixations on the mouth between the first and second fixation. This indicates the presence of an early bias during face processing, leading individuals with 22q11.2DS to direct their attention toward the mouth and to gradually increase their fixations on this specific feature. Similarly, several studies have already reported that participants with social anxiety disorder, social phobia, and schizophrenia are less likely to look at salient features when exploring faces [33, 34, 50–52]. Future research should further investigate the dynamic and the temporal evolution of the initial fixation positions to better understand the mechanisms underlying these particularities. Although individuals with 22q11.2DS tend to look more at the eyes compared to the mouth or nose, their scanpath remains atypical when compared to the TD group. Indeed, the proportion of time spent on mouth and features outside of the face region (shower cap) was greater than what was observed in the TD group. These results could indicate that participants with 22q11.2DS have difficulties to identify and maintain attention on socially relevant features (e.g., eyes) during face processing, which is line with previous research [13, 17]. Another possible interpretation for these findings is that an early bias toward the mouth and a return to the scanning of non-salient features occurring during the initial phase of face processing impair an optimal information extraction in participants with 22q11.2DS. These results have implications for the design of socio-emotional intervention programs aimed at improving efficiency in visual exploration and correcting face scanning from the very first fixations in individuals with developmental delay [53, 54]. Using cueing techniques, either explicit (e.g., a verbal cue) or implicit (e.g., a cross on the screen), may be one way to correct the bias to the mouth and improve sensitivity to configural changes in salient facial features. Further, targeting CFP may allow us to indirectly improve emotion and face recognition [23, 24]. For example, Russell et al. [55] found that verbal instructions during an emotional training task with patients with schizophrenia improved their emotion recognition by re-directing their attention to relevant facial features. Hadjikhani et al. [56] showed that visual cueing guiding the attention of individuals with ASD to the faces activates fusiform face area (FFA), a brain region specialized for face perception. However, these studies did not investigate whether the observed improvements in face processing after intervention are associated with changes in social functioning. Further work is therefore required to address this question more in depth.

Furthermore, participants with 22q11.2DS demonstrated longer fixation durations than the comparison group, confirming results from previous research [17]. Longer fixation duration is usually described as a marker of higher cognitive load and may thus indicate a more effortful information processing [29]. Our results also showed a reduced number of transitions between the two faces in the 22q11.2DS group. Consequently, we can assume that simultaneously exploring and comparing two faces was more difficult for participants with 22q11.2DS and involved more cognitive resources [17], which may lead to a perseverative and a less organized exploration (see also [53]). McCabe et al. [17] also reported poor performances on a task depicting faces in participants with 22q11.2DS and suggested that cognitive inflexibility could explain failure in meeting task demands when looking at more complex stimuli, such as human faces. Given that no significant association between IQ scores and fixation durations or number of transitions was found, the obtained results could be specific to the processing of social stimuli.

Finally, eye-tracking data highlighted distinct scanning paths during the CD vs. FD trials in the TD group only. A possible interpretation for this finding is that individuals with 22q11.2DS failed to modulate their visual scanpaths in order to adapt to stimuli and task demands. Accordingly, McCabe et al. [17] also found evidence of maladaptive visual scanning when participants with 22q11.2DS were looking at faces vs. weather scenes. This finding may indicate a lack of consistency when scanning faces (more variability in the way faces are explored) due to inattention and/or a difficulty adapting the scanning strategy to the context [13, 17, 57].

The second aim of our study was to examine FFP and CFP between groups and the developmental trajectory of CFP. Even though identifying configural differences was more difficult for both groups than identifying featural differences, CFP was proportionally more difficult than FFP for individuals with 22q11.2DS. They showed a larger gap in accuracy between CFP and FFP performances relative to TD individuals. As expected, these results extended the findings of a previous report [13] to a broader age range. Moreover, in accordance with our hypothesis, the longitudinal analyses revealed that individuals with 22q11.2DS improved to a lesser extent than TD participants in configural processing over time. This finding confirms in a broad age range the results previously reported in a cross-sectional sample [13]. A pronounced gap between featural and configural face processing in our 22q11.2DS sample could commensurate with structural or functional alterations in related brain regions. To date, few neuroimaging studies have provided evidence regarding the presence of separate cerebral pathways for the processing of featural and configural information [58, 59]. For example, Renzi et al. [58] conducted a Transcranial Magnetic Stimulation (TMS) study using configural and featural stimuli from the Jane task in a sample of healthy young adults. They found that the right inferior frontal cortex was responsible for the configural processing of faces, whereas the left middle frontal gyrus played a role for featural processing of faces. Furthermore, fMRI studies showed that individuals with 22q11.2DS have reduced activity compared to controls in cerebral regions involved in social cognition while looking at emotional faces [60] and show alterations in frontal brain regions [61, 62]. Combining fMRI and eye-tracking would enrich our knowledge regarding the cerebral regions contributing to configural face processing deficits in 22q11.2DS. The correlations between eye-tracking measures and behavioral results (task performance) were also investigated, but no significant association between these variables was observed. Contrary to what could have been expected, an increased time spent and number of fixations on the mouth did not help individuals with 22q11.2DS to distinguish the differences between the portraits and obtain a similar performance to TD in FD trials. These observations raise important questions regarding the link between eye gaze and performance on tasks using face stimuli, and this topic was already debated in previous studies [63, 64]. Our results might suggest that the eye-tracking measures used in the current study are insufficient for understanding task performance. Other mechanisms such as idiosyncratic scanning strategies and encoding process of visual stimuli and visual memory of faces could also contribute to their performance [63, 65, 66]. Future research should consider the use of the Dynamic Scanning Index (reflecting the number of times that the eye gaze goes in and out of a core feature) that might better reflect task performance [64].

Finally, we observed that individuals with more negative symptoms were characterized by lower performance on CD trials. As CFP has been shown to be important not only for face recognition but also for emotion recognition [23, 24], it is likely that difficulty perceiving configural changes may alter the ability to perceive emotional face changes during social interactions. However, the association between CFP and negative symptoms is rather modest and should be interpreted with caution. Contrary to our expectations, none of the eye-tracking or behavioral measures correlated with the anxiety measures. This result can be explained by the choice of our measures. Due to the wide age range of our sample, the anxiety measures used in the current study were different and less exhaustive than the ones used in Glaser et al. [13]. Further work is necessary to better understand the link between face processing, psychopathology, and social problems in 22q11.2DS.

The present study has several limitations that should be considered when interpreting the results. First of all, we cannot conclude from this study whether the atypical developmental trajectory of configural processing is face-specific. To answer that question, the development of configural and featural processing should also be explored in non-face stimuli (see [67]). For example, Giersch et al. [68] found evidence for spatial processing impairments during a discrimination task involving geometric forms, which may be suggestive of a global deficit in the configural processing in 22q11.2DS. A previous fMRI study [69] found that participants with 22q11.2DS did not show face-specific responses in the fusiform gyrus, while responses related to objects (houses) were intact in parahippocampal gyrus and similar to TD. This finding suggested that participants with 22q11.2DS are characterized by face-specific processing difficulties, rather than deficits in basic visual processing. Although a recent study showed that visual perception and processing, particularly form perception, predict performance on facial identity recognition in 22q11.2DS [70], no study to date has explored the link between basic perceptual and spatial processing and CFP and FFP. In the present study, we examined correlations between CFP and FFP performance and IQ measures related to full-scale score and visual processing (POI and PIQ) but did not find any significant association. This could indicate that the mechanisms underlying CFP and FFP are specific to the processing of social stimuli, but this interpretation needs to be considered with caution. Given that low IQ is an intrinsic characteristic of individuals with 22q11.2DS, the group (22q11.2DS vs. TD) and IQ variables are confounded. Hence, the observed differences could also be the result of intellectual impairments. The inclusion of IQ as a covariate in the analyses could be misleading and conduct to a bias caused by overadjustment [71–73]. Further work is required to better address this topic. For instance, the inclusion of a group with non-syndromic intellectual disability in future studies could help to better understand the impact of IQ on the different types of face processing. Another limitation concerns the choice of stimuli. In daily life, variations in facial features are more complex and social interactions are based on dynamic changes in facial expressions, whereas the stimuli used in this study are limited to static portraits of one individual. Another observation regarding the choice of stimuli is that some of the modified faces have an unnatural appearance, despite the fact that they were created based on anthropomorphic norms to maintain a natural quality [26]. Finally, eye-tracking has several limitations that have been described by Bojko [29]. Briefly, eye-tracking technology detects the foveal vision (center of the retina) and does not give information about the periphery of the visual field. For instance, upright faces can be processed by extracting information out of the foveal area only [20]. Furthermore, eye-tracking does not provide information about why a person looks at a stimulus of interest, or whether they understand what they see. However, reliable reporting about scanning behavior after completing a task is difficult to obtain in youngsters with developmental disabilities. Despite these drawbacks, eye tracking remains a useful technique that allows the investigation of scanning patterns and perception biases in clinical populations [74].

## Conclusions

Our study provides new evidence for the presence of atypical development and abnormal scanpaths during face processing in 22q11.2DS. The present findings are commensurate with previous results and uncover new aspects of the atypical face exploration observed in individuals with 22q11.2DS, providing a more complete picture of this specific issue. The current work is not only a larger follow-up of a previous report [13] but also raises new questions and paves the way for more research on the development of face processing and the clinical implications of perception biases present during face processing tasks in individuals with 22q11.2DS.

## Abbreviations

22q11.2DS: 22q11.2 deletion syndrome
ABCL: Adult Behavior Checklist
AOI: Areas of interest
ASD: Autism spectrum disorder
CBCL: Child Behavior Checklist
CD: Configural Different
CFP: Configural face processing
DICA-R: Diagnostic Interview for Children and Adolescents—Revised
FD: Featural Different
FFP: Featural face processing
FSIQ: Full Scale Intelligence Quotient
IQ: Intelligence quotient
K-SADS-P/L: Schedule for Affective Disorders and Schizophrenia for School-Age Children-Present and Lifetime Version
PANSS: Positive and Negative Syndrome Scale
PIQ: Performance Intelligence Quotient
POI: Perceptual Organization Index
PSI: Processing Speed Index
SCID-I: Structured Clinical Interview I
TD: Typically developed
VIQ: Verbal Intelligence Quotient
WAIS-III: Wechsler Adult Intelligence Scale—Version III
WISC-III: Wechsler Intelligence Scale for Children—Version III
